# Prognostic value of miR-142 in solid tumors: a meta-analysis

**DOI:** 10.1042/BSR20204043

**Published:** 2021-02-26

**Authors:** Rongqiang Liu, Shiyang Zheng, Kang Yu, Yajie Yu, Chenyu Yu, Wenqing Shi, Qianmin Ge, Zhiwei Ye, Yi Shao

**Affiliations:** 1 Department of Ophthalmology, The First Affiliated Hospital of Nanchang University, Nanchang 330006, Jiangxi, China; 2 Department of Hepatobiliary Surgery, The First Affiliated Hospital of Guangzhou Medical University, Guangzhou 510220, Guangdong, China; 3 Department of Breast Surgery, The Third Affiliated Hospital of Guangzhou Medical University, Guangzhou 510150, China; 4 Department of Gastrointestinal Surgery, The First Affiliated Hospital of Guangzhou Medical University, Guangzhou 510220, China

**Keywords:** cancer, meta-analysis, miR-142, prognosis

## Abstract

Several studies on the prognostic value of microRNA 142 (miR-142) in solid tumors have reported conflicting results. Therefore, the aim of this meta-analysis was to evaluate the relationship between the miR-142 and prognosis in solid tumors. A comprehensive search for relevant studies was conducted until 10 November 2020. Studies that investigated the prognostic significance of the miR-142 in solid tumors were included. The hazard ratio and 95% confidence interval were calculated using a random-effects model. All data analyses were performed using the STATA 12.0 software (Stata Corporation, College Station, TX, U.S.A.). Twenty articles involving 2451 participants were included in the meta-analysis. The results showed that high miR-142 expression was a better predictor of overall survival (OS) (HR = 0.66, 95% CI: 0.47–0.93) and disease-free/progression-free/recurrence-free survival (DFS/PFS/RFS) (HR = 0.71, 95% CI: 0.55–0.91) compared with low miR-142 expression. MiR-142 can be used as an effective prognostic marker for patients with solid tumors. Future large prospective studies are warranted to further confirm the present findings.

## Introduction

Globally, tumors have become a major disease threatening people’s health. Although great progress has been made in the prevention and treatment of cancer with the continuous progress in medicine, its morbidity and mortality continue to increase annually. The Global Cancer Observatory reported 18.1 million new cancer cases and 9.6 million cancer cases worldwide in 2018 [1]. The discovery of effective diagnostic and predictive markers may be one of the reliable clinical methods to overcome this major problem. MicroRNAs (miRNAs) are a class of small non-coding single-stranded RNAs (length: 20–24 nucleotides) regulating gene expression by binding to the 3′-untranslated region of the target mRNA [2]. In 2008, miRNA was first reported as a potential biomarker for tumors [3]. Since then, numerous studies have successively found that miRNAs are effective diagnostic and prognostic markers for a variety of tumors.

MiRNA-142 (miR-142), one of the important members of the miRNA family, was first discovered in chromosome 17 of hematopoietic stem cells. The miR-142 includes miR-142-3p and miR-142-5p, which are processed from the 3′ end and 5′ end of their precursors, respectively. Analysis of miR-142-3p or miR-142-5p provides the same effect as miR-142. MiR-142 is considered a tumor suppressor and is abnormally expressed in many tumors. Studies revealed that miR-142 participates in tumor cell proliferation, differentiation, invasion, and metastasis by regulating numerous genes, such as phosphatidylinositol-4,5-bisphosphate 3-kinase (PIK3CA), ras-related protein rap-1 A (RAP1A), Frizzled7 receptor (FZD7) and phosphatase and tensin homolog (PTEN) [4–7]. Recently, studies reported that the abnormal expression of miR-142 miRNAs is associated with the prognosis and clinical characteristics of many tumors, including hepatocellular carcinoma (HCC), colorectal cancer (CRC), non-small cell lung cancer (NSCLC), cervical cancer (CC), nasopharyngeal carcinoma (NPC), endometrial cancer (EC), esophageal squamous cell carcinoma (ESCC), renal cell carcinoma (RCC), intrahepatic cholangiocarcinoma (ICC), gastric cancer (GC), oral squamous cell carcinoma (OSCC) [7–26]. However, their results were inconsistent. Most investigations concluded that low miR-142 expression indicates a poor prognosis, whereas several other studies have shown that low miR-142 expression is beneficial to prognosis.

At present, the prognostic value of the miR-142 in tumors remains unclear. In the present study, we systematically searched for relevant articles and conducted a meta-analysis to better assess the relationship between the miR-142 family and survival in patients with cancer.

## Methods

### Search strategy

The PubMed, Embase, Web of Science, China National Knowledge Infrastructure and WanFang databases were systematically searched for relevant studies. The following keywords were used: ‘miR-142’ or ‘microRNA-142’ or ‘miRNA-142’ and ‘cancer’ or ‘carcinoma’ or ‘tumor’ or ‘tumour’ or ’neoplasm.’ Articles were searched until 1 November 2020. There were no language restrictions, and a manual search for articles in the reference lists of the included publications was also performed.

### Study selection

All articles were assessed independently by two investigators (Rongqiang Liu and Shiyang Zheng). Disagreements between these two investigators were resolved through discussion with a third investigator (Kang Yu). The following content criteria were used to select studies for inclusion in the analysis: (1) evaluation of miR-142 expression with survival outcomes in solid tumors; (2) availability of a hazard ratio (HR) with 95% confidence interval (95% CI); and (3) detection of miR-142 expression in human tumor tissue or serum. The exclusion criteria were: (1) insufficient data to calculate HRs with 95% CIs; (2) case reports, reviews, letters, conference papers, and animal experiments; (3) research data derived from public databases; and (4) duplicated or overlapped studies.

### Data extraction and quality assessment

The following relevant information was extracted: author name, publication year, country, study design, sample size, HR with 95% CI, miRNA types, detected sample, detection method, and HRs with 95% CIs for survival outcomes including overall survival (OS), disease-free survival (DFS), recurrence-free survival (RFS), and progression-free survival (PFS). We preferred to use the results of a multiple factor analysis owing to its high accuracy. In addition, for publications from which data could not be directly extracted, we obtained survival results from the survival curve according to Tierney et al.’s method [27]. The Newcastle–Ottawa Quality Assessment Scale (NOS) was used to evaluate article quality [28]. The present study did not require approval by the ethics committee.

### Statistical analysis

HR and corresponding 95% CI were used to analyze pooled data. A forest plot was used to explore the correlation between the miR-142 and prognosis in solid tumors. Heterogeneity analysis was performed using a chi-squared test or a Cochran’s Q test. Heterogeneity was assessed using the *I^2^* statistic. *I^2^* < 50% indicated minor heterogeneity and a fixed-effects model was used, while *I^2^* > 50 indicated substantial heterogeneity and a random-effects model was used. Subgroup sensitivity analyses were carried out to explore the sources of heterogeneity. Begg’s and Egger’s tests were used to analyze publication bias. Sensitivity analysis was used to test the stability of the results. All data analyses were performed using the STATA 12.0 software (Stata Corporation, College Station, TX, U.S.A.), and *P*<0.05 denoted statistically significant difference.

## Results

### Literature search

**Figure 1:**
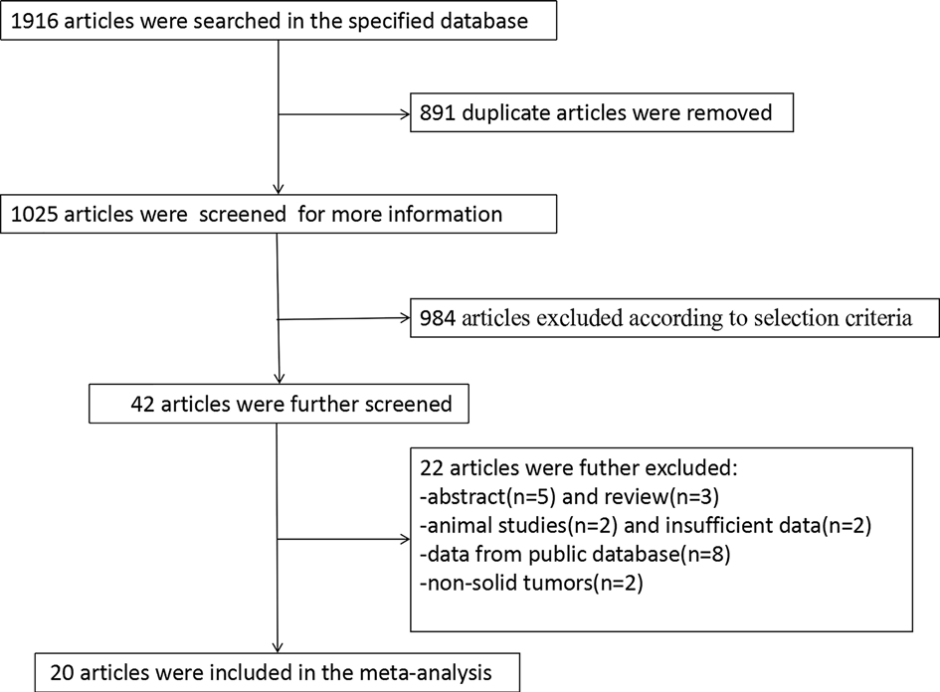
Flow chart of the article search

The literature search flow chart is shown in Figure 1. A total of 1916 articles were initially retrieved from the specified databases. After deleting the duplicates and those that did not meet the inclusion criteria or lacked data, a total of 20 articles were finally included in this meta-analysis. The included articles were published between 2011 and 2020. Fifteen and five articles were published in English [7–20,23] and Chinese [21,22,24–26], respectively.

### Study characteristics

The total number of patients included in the study was 2451 (range: 30–363 per study). The included studies were conducted in China, Germany and Australia. Five and fifteen articles detected the expression of miR-142 in blood and tissues, respectively. A total of 12 solid tumors were included, namely HCC [8,10], CRC [9], NSCLC [11,24], CC [12,15,21,26], NPC [13], EC [18], ESCC [14,22], RCC [16], glioma [17], ICC [7], GC [19,20,], melanoma [23] and OSCC [25]. The average NOS score of the included articles was 6.40. The basic information of the included studies is summarized in Table 1.

**Table 1:** The basic information of eligible studies

Study	Country	Cancer type	Sample	MiRNA type	Sample source	Survival outcome	NOS score
Chai, 2014	China	HCC	43	miR-142-3p	Tissue	DFS	7
Gao, 2019	China	CRC	363	miR-142-3p	Serum	OS	7
He, 2018	China	HCC	92	miR-142-3p	Tissue	OS	7
Kaduthanam, 2013	Germany	LC	204	miR-142-3p	Serum	RFS	7
Li, 2017	China	CC	173	miR-142-3p	Tissue	OS, PFS	7
Li, 2019	China	NPC	30	miR-142	Tissue	OS	7
Lin, 2011	China	ESCC	91	miR-142-3p	Tissue	OS	7
Lu, 2017	China	CC	85	miR-142-3p	Serum	OS, DFS	7
Peng, 2019	China	RCC	284	miR-142-3p	Tissue	OS	7
Qin, 2017	China	Glioma	97	miR-142	Tissue	OS	7
Su, 2018	China	EC	49	miR-142	Tissue	OS	7
Wei, 2019	China	ICC	100	miR-142-5p	Serum	OS	7
Yan, 2018	China	GC	101	miR-142-5p	Tissue	OS, RFS	6
Zhang, 2011	China	GC	65	miR-142-5p	Tissue	OS	6
Li, 2020	China	CC	173	miR-142-3p	Tissue	OS; DFS	5
Xu, 2018	China	ESCC	100	miR-142-5p	Tissue	PFS	5
Liu, 2017	China	LC	121	miR-142-3p	Tissue	OS	5
Zhang, 2020	China	OSCC	72	miR-142-3p	Tissue	OS	5
Su, 2020	China	CC	142	miR-142-3p	Serum	OS; PFS	5
Tembe, 2015A	Australia	Melanoma	66	miR-142-3p	Tissue	OS	7
Tembe, 2015B	Australia	Melanoma	66	miR-142-5p		OS	7

Abbreviation: LC, lung cancer.

### Association between miR-142 expression and OS

**Figure 2:**
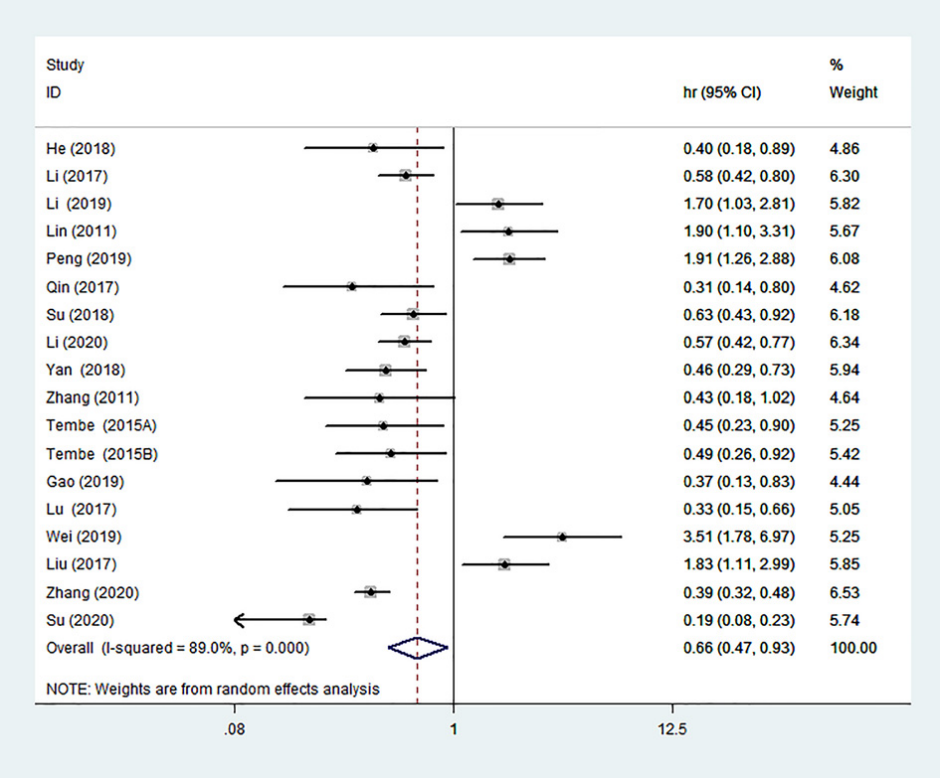
Forest plot of the association between high miR-142 expression and OS

**Figure 3:**
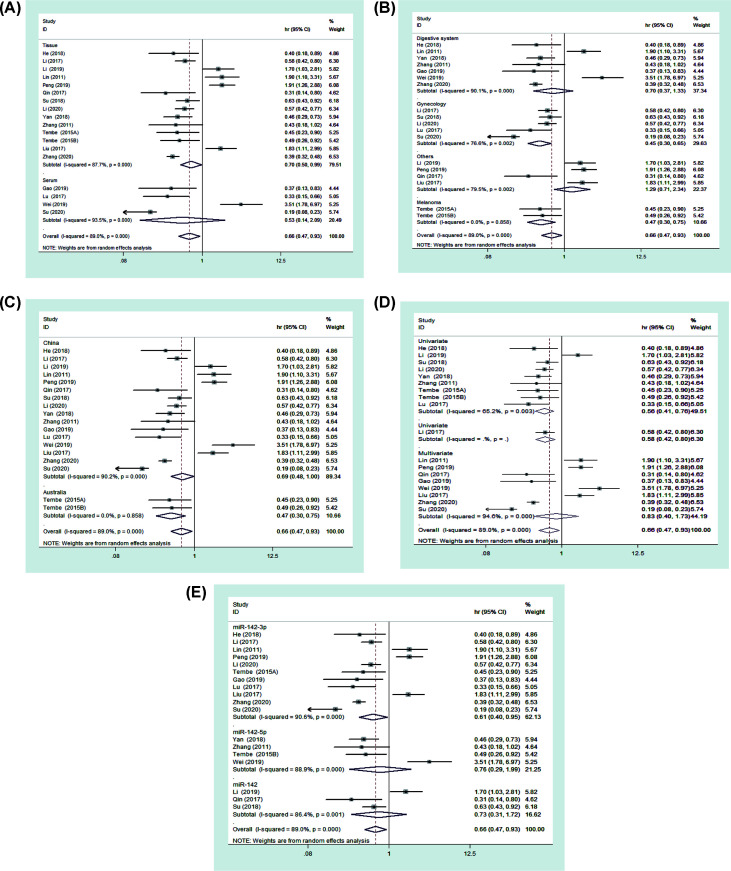
Forest plots of subgroup analysis of OS (**A**) The merged analyses in different sample groups. (**B**) The merged analyses in different cancers groups. (**C**) The merged analyses in different country groups. (**D**) The merged analyses in different analysis groups. (**E**) The merged analyses in different detection methods of miR-142.

Sixteen studies investigated the relationship between miR-142 expression and OS. Comprehensive analysis showed that there was significant relationship between high miR-142 expression and favorable OS (HR = 0.66, 95% CI: 0.47–0.93) (Figure 2). In order to further test the prognostic value of miR-142, we conducted the subgroup analysis based on cancer type, analysis type, country, detected sample, and detection of miR-142. Subgroup analysis revealed that high miR-142 expression indicated better OS in the subgroups of tissue (Figure 3A), gynecological tumors (Figure 3B), melanoma (Figure 3B), country (Australia or China) (Figure 3C), univariate (Figure 3D), methods identifying miRNAs (miR-142-3p) (Figure 3E). In addition, we also found that high miR-142 expression indicated better OS in GC (HR = 0.45, 95% CI: 0.30–0.68) and CC (HR = 0.40, 95% CI: 0.25–0.65). The results are presented in Table 2.

**Table 2:** Subgroup analysis for OS

Stratified analysis	Number of studies	Pooled HR (95% CI)	*P*-value	Heterogeneity		
				*I²* (%)	*P*-value	Model
Cancer type						
Digestive system	7	0.70 (0.37–1.33)	0.277	90.1	0	Random
GC	2	0.45 (0.30–0.68)	0	0	0.893	Fixed
Gynecology	5	0.45 (0.30–0.65)	0	76.6	0.002	Random
CC	4	0.40 (0.25–0.65)	0	80.5	0.002	Random
Melanoma	2	0.47 (0.30–0.75)	0.001	0	0.301	Fixed
Others	4	1.29 (0.71–2.34)	0.396	79.5	0.002	Random
Analysis type						
Univariate	10	0.57 (0.44–0.73)	0	60.9	0.006	Random
Multivariate	8	0.83 (0.40–1.73)	0	94.6	0	Random
Country						
Australia	2	0.47 (0.30–0.75)	0.001	0	0.505	Fixed
China	16	0.69 (0.48–1.00)	0.047	90.2	0	Random
Detected sample						
Tissue	14	0.70 (0.50–0.99)	0.042	87.7	0	Random
Serum	4	0.53 (0.14–2.09)	0.368	93.5	0	Random
Detection of miR-142						
miR-142	3	0.73 (0.31–1.72)	0.471	86.4	0	Random
miR-142-3p	11	0.61 (0.40–0.95)	0.027	90.6	0	Random
miR-142-5p	4	0.76 (0.29–1.99)	0.581	88.9	0	Random

### Association between miR-142 expression and DFS/PFS/RFS

**Figure 4:**
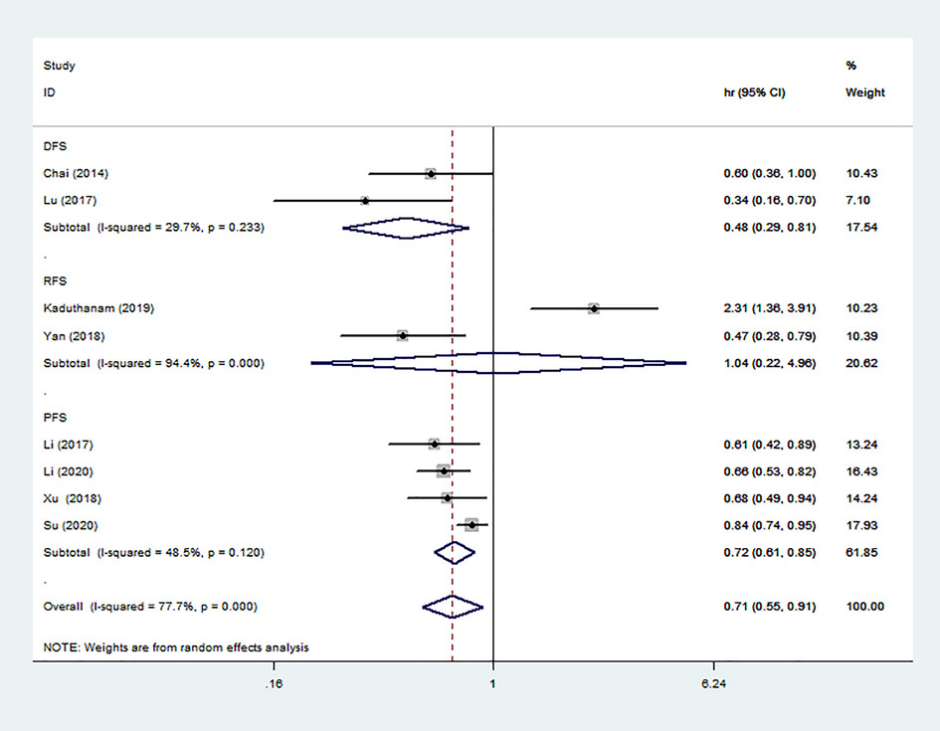
Forest plot of the relationship between high miR-142 expression and DFS/PFS/RFS

Eight studies reported a relationship between high miR-142 expression and DFS/PFS/RFS. Due to the obvious heterogeneity, the random effects model was adopted. The combined analysis suggested a significant relationship between high miR-142 expression and favorable DFS/PFS/RFS (HR = 0.71, 95% CI: 0.55–0.91) (Figure 4). Moreover, we found that high miR-142 expression also indicated favorable DFS (HR = 0.48, 95% CI: 0.29–0.81) and PFS (HR = 0.72, 95% CI: 0.61–0.85).

### Association between miR-142 expression and clinicopathological features

Through the analysis of the relationship between miR-142 expression and the clinical characteristics of tumor (Table 3), we found that high miR-142 expression was obviously associated with tumor stage (III–IV vs I–II)(OR = 0.33, 95% CI: 0.17–0.64), lymph node status (Yes vs No) (OR = 0.29, 95% CI: 0.16–0.52) and invasion depth (T3+T4 vs T2+T1) (OR = 0.39, 95% CI: 0.26–0.58). This obvious relationship was not observed in these clinical features, such as gender (Male vs Female), age (Old vs Young), tumor diameter (Big vs Small), tumor differentiation (Poor vs Moderate/Well) and distant metastasis (Yes vs No). We speculated that miR-142 participated in tumor differentiation, invasion and metastasis to affect tumor progression.

**Table 3:** Association between high miR-142 expression and clinicopathological features

Clinicopathologic features	Number of studies	Estimated OR (95% CI)	*P*-value	Heterogeneity		
				*I^2^* (%)	*P*-value	Model
Gender (Male vs Female)	8	0.98 (0.78–1.23)	0.884	0	0.521	Fixed
Age (Old vs Young)	11	1.02 (0.84–1.25)	0.825	0	0.927	Fixed
Tumor diamter (Big vs Small)	7	0.80 (0.55–1.16)	0.241	60	0.02	Random
Tumor stage ((III–IV vs I–II)	6	0.33 (0.17–0.64)	0.001	82	0	Random
Tumor differentiation (Poor vs Moderate/Well)	3	0.65 (0.26–1.64)	0.362	82.4	0.023	Random
Lymph node status (Yes vs No)	5	0.29 (0.16–0.52)	0.000	54.3	0.067	Random
Distant metastasis (Yes vs No)	2	2.38 (0.09–63.29)	0.604	93.3	0	Random
Invasion depth (T3+T4 vs T2+T1)	3	0.39 (0.26–0.58)	0	45.9	0.157	Fixed

### Sensitivity analysis

**Figure 5:**
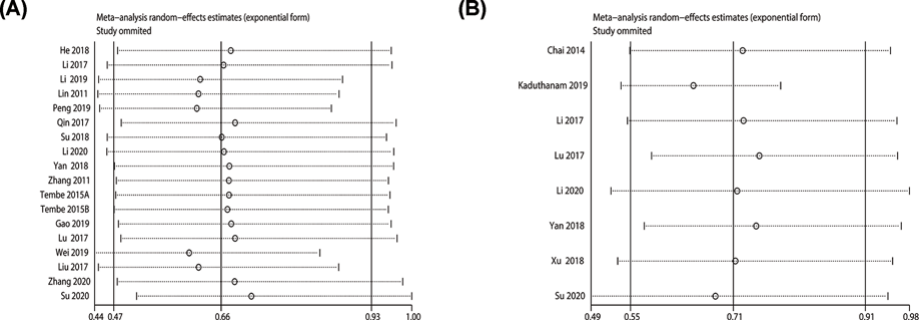
Sensitivity analysis (**A**) Sensitivity analysis for OS. (**B**) Sensitivity analysis for DFS/PFS/RFS.

Sensitivity analysis was implemented by excluding each study in turn. The results for OS (Figure 5A) or DFS/PFS/RFS (Figure 5B) did not differ significantly from those of the overall analysis, revealing that the outcomes were stable.

### Publication bias

**Figure 6:**
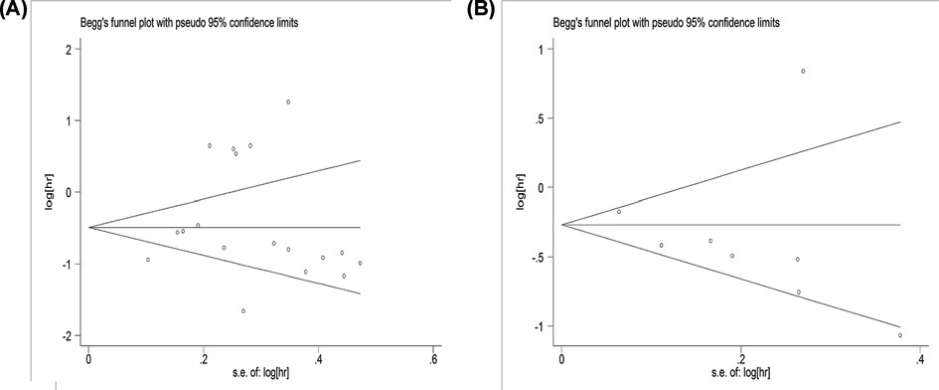
Funnel plots for publication bias (**A**) Funnel plots for OS. (**B**) Funnel plots for DFS/PFS/RFS.

Begg’s and Egger’s tests were applied to detect publication bias. In the analysis of association between high miR-142 expression and OS (Figure 6A), the *P*-values of Egger’s and Begg’s tests were 0.420 and 0.405, respectively. Regarding DFS/PFS/RFS (Figure 6B), the *P*-values were 0.480 and 0.386, respectively. All *P*-values were >0.05, indicating that there was no publication bias.

## Discussion

It has been confirmed that the expression of miR-142 is involved in tumor proliferation, metastasis, invasion and apoptosis [29]. Several studies have discussed the prognostic value of miR-142 expression in solid tumors. In HCC, He et al. reported that patients with high miR-142 expression had longer OS than those with low expression [10]. Similarly, in CC, Li et al. found that high miR-142 expression in blood was linked to better OS and PFS vs low expression [12]. However, other studies reported that high miR-142 expression was closely associated with poorer OS and RFS [11,14]. These conflicting results indicate that the miR-142 plays a dual role in cancer (tumor suppression and tumor promotion). The differences between studies may be caused by variations in research methods, statistical methods, detection methods, sample sizes and types, and the clinical experience of researchers. Although there are studies with inconsistent reports, it is undeniable that the miR-142 plays an important role in tumor progression.

Studies have shown that a cluster of miRNAs may be a better predictor of survival than a single miRNA [30]. In this study, we integrated all studies to evaluate the overall effect of the miR-142 on solid tumors prognosis. The combined results showed that high miR-142 expression was associated with favorable OS (HR = 0.66, 95% CI: 0.47–0.93) and DFS/PFS/RFS (HR = 0.71, 95% CI: 0.55–0.91). Subgroup analysis showed that high miR-142 expression mainly displayed good OS in GC, CC and melanoma. It was possible that miR-142 had better predictive value for the three tumors. We also observed that high miR-142 expression was positively associated with tumor stage (III–IV vs I–II), lymph node status (Yes vs No) and invasion depth (T3+T4 vs T2+T1). These results demonstrated that the miR-142 may be a potential prognostic marker in solid tumors.

The miR-142 regulates tumor progression in a variety of ways. Isobe et al. found that miR-142 affects the progression of breast cancer by regulating the WNT signaling pathway [31]. Mansoori et al. reported that miR-142 can target Bach-1 in breast cancer cells, thereby down-regulating the expression of C–X–C chemokine receptor type 4 (CXCR4), matrix metalloproteinase 9 (MMP9) and vascular endothelial growth factor receptor (VEGFR) [32]. This effect leads to inhibition of breast cancer cell proliferation, invasion and migration [32]. Shen et al. revealed that transfection of miR-142-3p mimics in colon cancer cells down-regulated the expression of cyclin D1 (CCND1), induced cell cycle arrest at the G_1_ phase, and increased the sensitivity of cells to 5-fluorouracil [33]. A potential mechanism of these miR-142-3p mimics is the suppression of tumor growth by down-regulating the expression of CD133, leucine-rich repeat containing G protein-coupled receptor 5 (LGR5), and ATP-binding cassette subfamily G member 2 (ABCG2) in colon cancer cells [33]. In lung cancer, miR-142 inhibited tumor cell proliferation and migration through metastasis associated lung adenocarcinoma transcript 1 (MALAT1)/-catenin signaling [34]. Cheng et al. suggested that miR-142 targets PLA2G16 through the extracellular signal-regulated kinase 1/2 (ERK1/2) signaling pathway to inhibit the proliferation of osteosarcoma cells and promote their apoptosis [35]. He et al. found that the taurine up-regulated 1 (TUG1)-miR-142-zinc finger E-box binding homeobox 1 (ZEB1)/epithelial–mesenchymal transition axis structure exists in HCC. miR-142 up-regulates ZEB1 by combining with the TUG1 to inhibit the proliferation, migration, invasion and epithelial–mesenchymal transition of hepatocarcinoma cells [10]. Zhu et al. displayed that the miR-142 mimic reduced *in vitro* cell viability and colony formation by inducing cell cycle arrest in CRC-derived cells, and inhibited* in vivo* tumor cell growth in xenografted nude mice [36]. This mimic may perform its biological function by regulating CDK4 cells [36]. Based on the above evidence, miR-142 has different regulatory mechanisms in the tumorigenesis of various types of cancer and may be a potential target for anti-tumor therapy.

This meta-analysis was the first to investigate the prognostic value of the miR-142 in solid tumors. Nevertheless, there are many limitations in the present study. Firstly, some results extracted from the survival curve may not accurately reflect the true values. Secondly, all included studies involved small samples, and the statistical power of each study was limited. Thirdly, there was obvious heterogeneity for OS, and we found that tumor types may be the source of heterogeneity. Fourthly, the sensitivity analysis for OS showed that the results were unstable. We believe that this may be due to the use of different research methods, differences in research quality, statistical methods, and adjustment factors. Fifthly, most of the research was conducted in China, which may affect the generalizability of the findings. In addition, we did not evaluate the relationship between the miR-142 and other clinicopathological parameters. Finally, we did not evaluate the prognostic value of miR-142 combined with other miRNA markers.

In summary, our study confirmed that high miR-14 expression predicted favorable OS and DFS/PFS/RFS. MiR-142 can be used as an effective prognostic marker for patients with solid tumors. Additional large, prospective, high-quality studies are warranted to confirm the present findings and fully clarify the potential function of the miR-142 in the progression of cancer.

## Data Availability

All data are in the manuscript and can be obtained from the corresponding authors.

## Abbreviations

CC: cervical cancer
CI: confidence interval
CRC: colorectal cancer
DFS: disease-free survival
EC: endometrial cancer
ESCC: esophageal squamous cell carcinoma
GC: gastric cancer
HCC: hepatocellular carcinoma
HR: hazard ratio
ICC: intrahepatic cholangiocarcinoma
miRNA: microRNA
NOS: Newcastle–Ottawa Quality Assessment Scale
NPC: nasopharyngeal carcinoma
NSCLC: non-small cell lung cancer
OS: overall survival
OSCC: oral squamous cell carcinoma
PFS: progression-free survival
RCC: renal cell carcinoma
RFS: recurrence-free survival
TUG1: taurine up-regulated 1
ZEB1: zinc finger E-box binding homeobox 1
